# Antioxidant, Hepatoprotective Potential and Chemical Profiling of Propolis Ethanolic Extract from Kashmir Himalaya Region Using UHPLC-DAD-QToF-MS

**DOI:** 10.1155/2015/393462

**Published:** 2015-10-11

**Authors:** Adil F. Wali, Bharathi Avula, Zulfiqar Ali, Ikhlas A. Khan, Ahlam Mushtaq, Muneeb U. Rehman, Seema Akbar, Mubashir Hussain Masoodi

**Affiliations:** ^1^Department of Pharmaceutical Sciences, University of Kashmir, Srinagar, Jammu and Kashmir 190006, India; ^2^National Center for Natural Products Research, Research Institute of Pharmaceutical Sciences, The University of Mississippi, University, MS 38677, USA; ^3^Division of Pharmacognosy, Department of BioMolecular Sciences, School of Pharmacy, The University of Mississippi, University, MS 38677, USA; ^4^Department of Biochemistry, University of Kashmir, Srinagar, Jammu and Kashmir 190006, India; ^5^Department of Biochemistry, Faculty of Veterinary Sciences and Animal Husbandry, Sher-e-Kashmir University of Agricultural Sciences and Technology of Kashmir, Jammu and Kashmir 190006, India; ^6^Central Council for Research in Unani Medicine, University of Kashmir, Srinagar, Jammu and Kashmir 190006, India

## Abstract

The aim of this study was to examine hepatoprotective effect of ethanolic extract of propolis (*KPEt*) from Kashmir Himalaya against isoniazid and rifampicin (INH-RIF) induced liver damage in rats. Hepatic cellular injury was initiated by administration of INH-RIF combination (100 mg/kg) intraperitoneal (i.p.) injection for 14 days. We report the protective effects of *KPEt* against INH-RIF induced liver oxidative stress, inflammation, and enzymatic and nonenzymatic antioxidants. Oral administration of *KPEt* at both doses (200 and 400 mg/kg body weight) distinctly restricted all modulating oxidative liver injury markers and resulted in the attenuation of INH-RIF arbitrated damage. The free radical scavenging activity of *KPEt* was evaluated by DPPH, nitric oxide, and superoxide radical scavenging assay. The components present in *KPEt* identified by ultra high performance liquid chromatography diode array detector time of flight-mass spectroscopy (UHPLC-DAD-QToF-MS) were found to be flavonoids and phenolic acids. The protective efficacy of *KPEt* is possibly because of free radical scavenging and antioxidant property resulting from the presence of flavonoids and phenolic acids.

## 1. Introduction

Tuberculosis (TB) is a foremost global health concern over years. According to World Health Organization (WHO) global tuberculosis reports, globally there are over 9 million people who develop TB each year and India has been classified on 8th rank among the 22 high burden countries [1]. Rifampicin (RIF) and isoniazid (INH) are the front line drugs that are used in the chemoprophylaxis and management of TB [2]. Previously published reports suggest that INH-RIF has potential of hepatic toxicity. Liver toxicity and hepatitis are presumed to be augmented with synergistic use of many medications including RIF and alcohol abuse. There is increased level of liver enzyme markers in serum (aspartate transaminase and alanine transaminase), fatal hepatitis, bilirubinemia, bilirubinuria, and jaundice, with dosing schedule consisting of INH and RIF. The common prevenient symptoms of hepatitis are anorexia, nausea, vomiting, fatigue, malaise, and weakness [3, 4]. Sarich et al. reported that administration of INH-RIF dose simultaneously in rabbits results in elevation of phospholipids and a reduction in phosphatidylcholine, cardiolipin, and inorganic phosphates, possibly via a choline deficiency, which may lead to the observed liver toxicity [5]. Further Zhang et al. observed that coadministration INH-RIF caused steatosis, increased apoptosis of the hepatocytes, and hepatic oxidative stress [6]. About 9.5% Indian patients have been reported to develop hepatotoxicity due to antitubercular therapy [7]. Therefore, there is requirement to investigate the natural molecules that can successfully diminish the toxicity to improve their chemotherapeutic efficacy. Nowadays, dietary supplements containing natural products, fruits, vegetables, medicinal plants, and herbs have many biological properties and have potential to fight against several human pathogens [8].

Propolis, also known as “*bee glue*,” is a resinous material produced by honeybees which they collect from different species of plants, to use it as beehive sealant. The chemical magnitudes of the propolis are influenced by various aspects such as geographical site, seasonal diversification, flora origin, and collection time (year), which is responsible for the diverse pharmacological activities of the propolis [9]. In the past, propolis has been used in folk remedy for several ailments and various pharmacological properties such as anti-inflammatory [10], antimicrobial [11], antioxidant [12], immunostimulant [13], antitumor [14], neuroprotective [15], and hepatoprotective activity [16] have been reported.

Our present study is the first approach to investigate and validate scientifically* in vitro *antioxidant potential followed by chemical profiling using UHPLC-DAD-QToF-MS and* in vivo *hepatoprotective activity of ethanolic extract of propolis from Kashmir Himalaya region.

## 2. Materials and Methods

### 2.1. Material Collection and Preparations of Propolis Ethanolic Extract (*KPEt*)

The propolis used for study was collected from Central Kashmir, Rangil, Ganderbal (Jammu and Kashmir, India), which was identified and authenticated from “Research and Training Center for Pollinator, Pollinizer and Pollination Management,” Sher-e-Kashmir University of Agricultural Sciences and Technology, Kashmir, India, under specimen voucher number AU/DR/NAE-II/137. The propolis was packed into percolator and extracted with 100% ethanol at room temperature with constant agitation for 24 hrs. After several cycles of extraction the extract was filtered and recovered under reduced pressure. The yield of* KPEt *was 33.37% w/w. The extract was then kept in desiccator to remove moisture and finally kept in refrigerator for further use.

### 2.2. Chemical Profiling of* KPEt* Using UHPLC-DAD-QToF-MS

Chemical profiling of* KPEt* was carried out on LC-MS instrument (Agilent QToF-MS 6530 series, Agilent Technologies, Palo Alto, CA, USA) coupled with Agilent UHPLC 1290 Series (Agilent Technologies, Palo Alto, CA, USA) with ESI interface. The LC-MS operating parameters were as follows: the spectra were obtained in ESI+ and ESI− modes, gas temperature 250°C, gas flow 10 L/min, nebulizer 30 psig, sheath gas temperature 325°C, capillary voltage 3.0 kV, and fragmentor 125 V. The chromatographic separation was achieved on ZORBAX SB-C18 RRHD 1.8 *μ* column (2.1 × 150 mm) at a flow rate of 0.25 mL/min with mobile phase (A) water and (B) acetonitrile, both containing 0.1% formic acid with gradient program as follows: A 85% : 15% B to A 45% : B 55% in 20 min and to 100% B in next 3 min. The column temperature was operated at 40°C and injection volume 2 *μ*L. DAD spectra were acquired over a scan range of 190–600 nm. All the operations, acquisition, and analysis of data were controlled by Agilent MassHunter Acquisition Software version A.05.00 and processed with MassHunter Qualitative Analysis Software version B.05.00. Each sample was analyzed in both positive and negative modes in the range of* m/z* = 100–1000. Accurate mass measurements were obtained by means of ion correction techniques using reference masses at* m/z* 121.0509 (protonated purine) and 922.0098 [protonated hexakis (1H, 1H, 3H-tetrafluoropropoxy) phosphazine or HP-921] in positive ion mode, while at* m/z* 112.9856 (deprotonated trifluoroacetic acid (TFA)) and 1033.9881 (TFA adducted HP-921) they were used in negative ion mode. The compounds were confirmed in each spectrum. For this purpose, the reference solution was introduced into the ESI source via a T-junction using an Agilent Series 1200 Isocratic Pump (Agilent Technologies, Santa Clara, CA, USA) using a 100 : 1 splitter set at a flow rate of 20 *μ*L/min.

### 2.3. Antioxidant Property

#### 2.3.1. 1,1-Diphenyl-2-picrylhydrazyl Radical Scavenging Activity (DPPH)

The free radical scavenging assay of* KPEt* was measured using a modified DPPH assay method by Huang et al. [20]. Aliquots of 0.3 mL of various concentrations (50–250 *μ*g/mL) of* KPEt* were mixed with a solution of 0.2 mmol/L DPPH in methanol (2.7 mL). The mixture was mixed vigorously, and absorbance value was recorded at 517 nm using UV-Spectrophotometer (Model UVD-2950, Labomed Inc.) after incubation at room temperature for 15 min in dark. The percentage of radical scavenging activity is determined using the following formula:

(1) $$\begin{array}{c} Radical\;\;scavenging\;\;activity\;\;\%=\left[\;\frac{A_{C}-A_{S}}{A_{C}}*100\;\right], \end{array}$$

where *A*_C_ is the absorbance of DPPH without sample and *A*_S_ is the absorbance of the DPPH with* KPEt*/vitamin C. All the samples were investigated in triplicate.

#### 2.3.2. Nitric Oxide Radical Scavenging Assay

Nitric oxide radical inhibition was estimated using Griess Ilosvay reaction [21]. In this investigation, Griess Ilosvay reagent was generally modified by using naphthyl ethylenediamine dihydrochloride (0.1% w/v) instead of 1-naphthylamine (5%). The reaction mixture (3 mL) containing 2 mL of 10 mM sodium nitroprusside, dissolved in 0.5 mL saline phosphate buffer (pH 7.4), is mixed with 0.5 mL of* KPEt* at various concentrations (50–250 *μ*g/mL) and was incubated at 25°C for 150 minutes. After incubation, 0.5 mL of the incubated aliquots is withdrawn and mixed with 0.5 mL of Griess reagent [1.0 mL sulfanilic acid reagent, 0.33% in 20% glacial acetic acid] at room temperature for 5 min with 1 mL of naphthyl ethylenediamine dichloride (0.1% w/v). The mixture is then incubated at room temperature for 30 min and its absorbance value was recorded at 546 nm using UV-Spectrophotometer (Model UVD-2950, Labomed Inc.).

The percentage of nitric oxide scavenging activity is determined using the following formula:

(2) Nitric  Oxide  Scavenging  Activity  %=AC−ASAC∗100,

where *A*_C_ is the absorbance of mixture of Griess reagent and naphthyl ethylenediamine dihydrochloride and *A*_S_ is the absorbance of mixture of Griess reagent and naphthyl ethylenediamine dihydrochloride solution with* KPEt*/chrysin. All the samples were investigated in triplicate.

#### 2.3.3. Superoxide Radical Anion Scavenging Assay

The superoxide anion radical scavenging activity was investigated by riboflavin-light-NBT system [22]. In this assay the reaction mixture had 0.5 mL of 50 mM phosphate buffer (pH 7.6), 0.3 mL riboflavin (50 mM), 0.25 mL phenazine methosulphate (20 mM), and 0.1 mL nitro blue tetrazolium (0.5 mM), before 1 mL* KPEt *solution was added at various concentrations (50–250 *μ*g/mL). Reaction commenced as the reaction mixture was illuminated with different concentrations of the* KPEt* using a fluorescent lamp. After 20 min of incubation, the absorbance values were recorded at 560 nm using UV-Spectrophotometer (Model UVD-2950, Labomed Inc.). The percentage of superoxide scavenging activity was calculated as follows:

(3) Superoxide  scavenging  capability  %=AC−ASAC∗100,

where *A*_C_ is the absorbance of the reaction mixture without* KPEt*/quercetin and *A*_S_ is the absorbance of the reaction mixture with* KPEt*/quercetin. All the samples were investigated in triplicate.

### 2.4. Evaluation of Hepatoprotective Activity

#### 2.4.1. Experimental Animals

Male Wistar albino rats (8–10 weeks old) were selected and used in the present experiment. The animals were housed in a group of six in polypropylene cages with saw dust as bedding in animal house facility of Department of Pharmaceutical Sciences, University of Kashmir. All the experiments were performed according to protocols authorized by CPCSEA animal ethical committee; the animal studies had approval of IAEC, Department of Pharmaceutical Sciences, University of Kashmir, under project number F-IAEC (Pharm. Sc.) APPROVAL/2013/21, dated September 28th, 2013. The animals were maintained under exposure to a 12 h/12 h light/dark cycle at a room temperature of 22–24°C and free access to standard laboratory feed (M/s Ashirwad Industries, Mohali, India) and water* ad libitum*.

#### 2.4.2. Acute Toxicity Testing

The acute toxicity study was performed as per Organization for Economic Cooperation and Development [23]. Single dose of* KPEt* 2000 mg/Kg b.wt was orally administrated. Mortality, behavior activities, body weight, and food and water consumption were monitored for 14 days.

#### 2.4.3. Animal Study Design

The treatment execution for* KPEt* of propolis and the approach of verifying its hepatoprotective efficacy against hepatotoxins was based on the prelude dose dependent pilot study. All rats were divided into five groups of six rats each. Selection of the dose course was based on acute toxicity study. The following treatment regimen was followed for 14 days study [24]:

- Group I: only normal saline, 0.9% p.o.
- Group II: INH-RIF 100 mg/kg b.wt i.p.
- Group III: INH-RIF 100 mg/kg b.wt i.p. + Silymarin 100 mg/kg b.wt p.o.
- Group IV: INH-RIF 100 mg/kg b.wt i.p. +* KPEt* 200 mg/kg b.wt p.o.
- Group V: INH-RIF 100 mg/kg b.wt i.p. +* KPEt* 400 mg/kg b.wt p.o.

At the end of the study, rats were sacrificed by cervical dislocation under mild anesthesia and blood was taken by dorsal vena cava for various serological parameters. Liver was dissected out and used for* in vivo* antioxidant studies, histological and immunohistochemistry examination.

#### 2.4.4. Estimation of Serum Biochemistry

The hepatoprotective potential of* KPEt* was studied by assessing the levels of sera ALT, AST, ALP, TP, T. bil, CHL, and TG using assay kit (Accurex Biomedical kits, Mumbai, India) according to the manufacturer's protocol on RIELE photometer 5010 V5+ semi-auto-analyzer (Berlin, Germany).

#### 2.4.5. Determination of* In Vivo* Antioxidant Enzyme Activities

The liver tissues were homogenized and were used for various* in vivo* antioxidant enzyme assays such as GPx, MDA, XO, SOD, and CAT [25].

#### 2.4.6. Histological Examination

The wet livers tissues were fixed in formalin and dehydrated; a section of liver 5 *μ*m was cut and stained with haematoxylin and eosin (H&E) dye for examination. Then these slides were investigated for histopathological alteration under fluorescent microscope BX-100 (Olympus Life Science, Europa GMBH, Wendenstrasse 14-18, 20097 Hamburg, Germany).

#### 2.4.7. Immunohistochemistry Examination

The liver tissues were fixed in formalin and embedded in paraffin. Sections of 5 mm thickness were cut onto polylysine coated glass slides. Sections were deparaffinized three times (5 min) in xylene followed by dehydration in graded ethanol and finally rehydrated in running tap water. For antigen retrieval, sections were boiled in 10 mM citrate buffer (pH 6.0) for 5–7 min. Sections were incubated with hydrogen peroxide for 15 min to minimize nonspecific staining and then rinsed three times (5 min each) with 1x PBST (0.05% Tween 20). Blocking solution was applied for 10 min; then sections were incubated with diluted (1 : 100 for NF-*κ*B and COX-2) primary antibodies, purified rabbit polyclonal anti-NF-*κ*B antibody (BioLegend), and rabbit polyclonal anti-COX-2 antibody (BioVision), overnight at 4°C in humid chamber. Further processing was done according to the instructions of UltraVision plus Detection System Anti-Polyvalent, HRP/DAB (Ready-To-Use) staining kit (Thermo Scientific system). The peroxidase complex was visualized with 3,30-diaminobenzidine (DAB). Lastly the slides were counterstained with hematoxylin, cleaned in xylene, and dehydrated with ethanol and after DPX mounting microscopic (BX51 Olympus) analysis was done at 400x magnification [26].

#### 2.4.8. Quantitative Evaluation of NF-*κ*B and COX-2 Immunostaining

According to the diffuseness of the DAB staining, sections were graded as 0 (no staining), 1 (staining, 25%), 2 (staining between 25% and 50%), 3 (staining between 50% and 75%), or 4 (staining >75%). According to staining intensity, sections were graded as follows: 0 (no staining), 1 (weak but detectable staining), 2 (distinct staining), or 3 (intense staining). Immunohistochemical staining scores were obtained by adding the diffuseness and intensity scores. All slides were examined by two independent observers who were unaware of the experimental protocol. The slides with discrepant evaluations were reevaluated, and a consensus was reached. Measurements were carried out using an Olympus BX51 microscope using objectives with 40x magnifications.

### 2.5. Statistical Analysis

All the results were expressed as mean ± S.E.M. Difference between the groups was analyzed by using one-way analysis of variance (ANOVA) followed by the Tukey-Kramer multiple comparison test using GraphPad prism software version 6.01 (GraphPad software, San Diego, USA).

## 3. Results and Discussion

**Figure 1:**
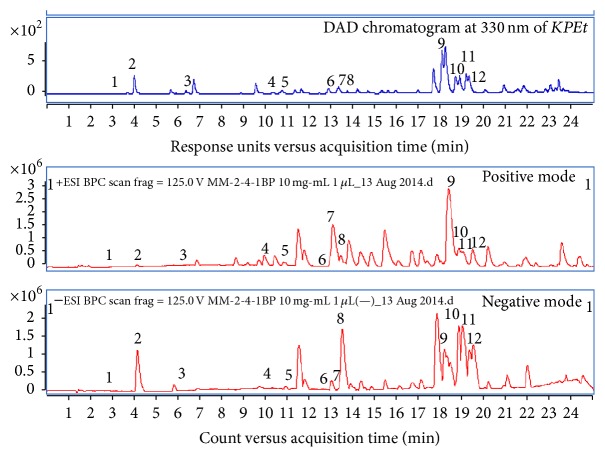
Representative chromatographic profile of* KPEt* at 330 nm and BPC in both positive and negative mode.

This work is proposed to investigate the antioxidant potential and hepatoprotective efficiency of ethanolic extract of the propolis from Kashmir region along with its chemical profiling using UHPLC-DAD-QToF-MS. Propolis contains various bioactive secondary metabolites mostly phenolic acids followed by flavonoids, which have been identified as potent antioxidants [27]. An investigation of* KPEt* was carried out by means of UHPLC-DAD-QToF-MS; the accurate mass and *λ*_max_ of the identified compounds were correlated with reference library, reference samples spectra, research papers, and online mass spectral based catalog like https://origin-scifinder.cas.org/scifinder/login and http://www.ncbi.nlm.nih.gov/pmc/. The compounds identified are hydroxycinnamic acid derivatives and flavonoids (flavones, flavonol, and flavanone derivatives) and the order of elution in both base peak chromatograms (BPC) positive and negative mode is shown in Figure 1; all the compounds have to be detected in both positive and negative modes. In (−)-ESI-MS, the mass spectra of the chromatographic peaks showed deprotonated molecules [M − H]^−^ and protonated molecules [M + H]^+^ in positive ion mode. The data provided in Table 1 is supported by the information acquired from the analyses represented in the TIC and UV chromatogram in Figure 1.

From the previous investigation it has been reported that presence of flavonoids and phenolic acids is responsible for hepatoprotective activity by decreasing the level of hepatic markers, lipid peroxidation, and attenuates free radical scavenging potential [28–30]. Flavonoids, particularly flavonols, possess various pharmacological activities that contribute to health benefits that include antioxidant and hepatoprotective activity [31]. Besides that flavonols also avert oxidative stress by direct scavenging of free radicals, metal chelation, and induction of antioxidant enzymes as well as phase II detoxifying enzymes [32].

DPPH assay is the most common assay used to determine the radical scavenging capacity of various compounds as it has ability to donate hydrogen to free radicals. The antioxidant property of* KPEt* was assessed by its potential to scavenge DPPH radical. The result in Table 2 depicted significant scavenging potential of* KPEt* with IC_50_ value of 52.16 ± 6.39 *μ*g/mL and the reference vitamin C showed scavenging potential of 11.28 ± 5.23 *μ*g/mL. The mechanism behind the radical scavenging property is because of presence of flavonoids and phenolics, which are mostly weak acids in nature, and therefore act as proficient electron donors able to react with O_2_^•−^ depending upon the substitution in the phenolic ring [33].

Superoxide radical anion scavenging assay of* KPEt* showed IC_50_ value of 34.77 ± 4.23 *μ*g/mL while chrysin (reference) showed IC_50_ value of 42.54 ± 5.10 *μ*g/mL. Superoxide radical anion (O_2_^•−^) is generated by number of metabolic processes and has ability to react with the cell and induce cellular damage and various diseases [34]. Antioxidant capacity of various flavonoids is primarily due to scavenging of superoxide anion [35]. As shown in Table 2, IC_50_ value of* KPEt* is less than that of the reference; therefore, the results revealed that* KPEt* is having stronger scavenging ability than the reference.

Nitric oxide assay of* KPEt* demonstrated moderate antioxidant potential in nitric oxide scavenging assay with IC_50_ value of 74.62 ± 5.23 *μ*g/mL, whereas the standard antioxidant quercetin (reference) showed IC_50_ value of 51.02 ± 3.63 *μ*g/mL. Nitric oxide radical (NO^•^) is essential in the regulation of various physiological and pathophysiological processes and is produced by specific nitric oxide synthases [36]. Nitric oxide radical reacts with superoxide radical anion (O_2_^•−^) and produces peroxynitrite anion (ONOO^−^) [33] which in physiological environment forms adduct with CO_2_ dissolved in the body fluids; this adduct is believed to be responsible for the oxidative damage [37]. The results in Table 2 suggest that the* KPEt* has less effective nitric oxide scavenging potential than the reference.

During acute toxicity testing of* KPEt* no adverse/toxic signs were observed showing nontoxic nature of* KPEt*. On the basis of the results of acute toxicity testing 1/10th (200 mg/Kg b.wt) and 1/5th (400 mg/Kg b.wt) dose were selected to be administered in rats throughout the experiment.

Silymarin is a natural compound isolated from* Silybum marianum*, which is commonly known as milk thistle. Silymarin is a flavonolignan extract, mainly containing flavonoid, including silibinin or silibinin, silydianin, and silychristin [38]. Silymarin has been found cure numerous liver disorders as it has traditionally restored the efficacy of liver function and regeneration of hepatic cells [39]. Moreover, it is used as a reference drug and showed evidence of potent liver protective activity within the dose range of 25 to 200 mg/kg [40]. It has already been established that in RIF and INH induced toxicity there is change in liver cellular fortification mechanisms, both enzymatic and nonenzymatic [41]. During the acetylation of INH by the liver enzyme N-acetyl transferase 2, acetylhydrazine and isonicotinic acid are produced. Further acetylhydrazine on hydrolysis produces hydrazine and diacetylhydrazine; both of these metabolites cause irremediable cellular injury [42]. RIF gets metabolized to desacetyl rifampicin in liver which on further hydrolysis forms 3-formyl rifampicin which is responsible for hepatocellular injury [43]. RIF is also a strong inducer of Cyt P_450_ when coadministrated with other antituberculosis drugs which lead to toxicity of liver [44]. The rodents were stressed with hepatoxin (INH-RIF) which induced hepatotoxicity and caused hepatic cellular injury which is comprised of centrilobular necrosis, hepatic cell augmentation, steatosis, and inhibition of endogenous antioxidants [45]. These in turn lead to the elevation of enzymes in serum due to the leakage of enzymes from liver (ALT, AST, ALP, and T.bil), increase in lipid profile (CHL, TG), and decrease in TP in blood. These liver enzymes in serum are constructive quantitative markers and nature of hepatic cell damage.  Table 3 signifies the effects of INH-RIF and* KPEt* (200 mg and 400 mg/Kg b.wt) on enzymatic markers. In group II there was considerable elevation in the levels of hepatic enzymes, lipid profile, and lowering in total protein content as compared to group I (*P* < 0.001). In groups III, IV, and V after the administration of* KPEt* and INH-RIF there were significant decrease in ALT, AST, ALP, T.bil, CHL, and TG content and elevation in TP when compared with group II (*P* < 0.001 and 0.01, resp.). Therefore, treatment regimen executed with groups III and V demonstrated remarkable hepatoprotective activity by bringing the enzyme levels and other biochemical parameters towards normal as compared with group II.

The* in vivo* antioxidant ability of* KPEt* was investigated using INH-RIF stressed hepatotoxicity and the results revealed that in group II there were significant (*P* < 0.001) decrease in the concentration of GPx, CAT, SOD, and XO and increase in MDA level when compared with group I (Table 4). Raised levels of free radicals and oxidative stress are related to the hepatopathy due to augmentation of free radicals and slackening of scavenging capacity of the hepatocytes [46]. After the treatment with* KPEt*, there was noticeable decrease in the levels of MDA in groups III, IV, and V when compared with group II (*P* < 0.001 and 0.01).* KPEt* at the dose of 400 mg/kg b.wt group V substantially decreased the lipid peroxidation as compared with 200 mg/kg b.wt dose group IV. In groups III, IV, and V the levels of GPx, CAT, SOD, and XO were considerably increased after the administration of* KPEt* at the dose of 400 mg/Kg b.wt and 200 mg/Kg b.wt as compared with group II (*P* < 0.001 and 0.01). The results acquired in the present study are consistent with the previous investigations where there are decrease in the levels of GPx, CAT, SOD, and XO and increase in MDA level by INH-RIF in comparison to the normal, revitalizing the attenuated scavenging capacity of the hepatocytes.

**Figure 2:**
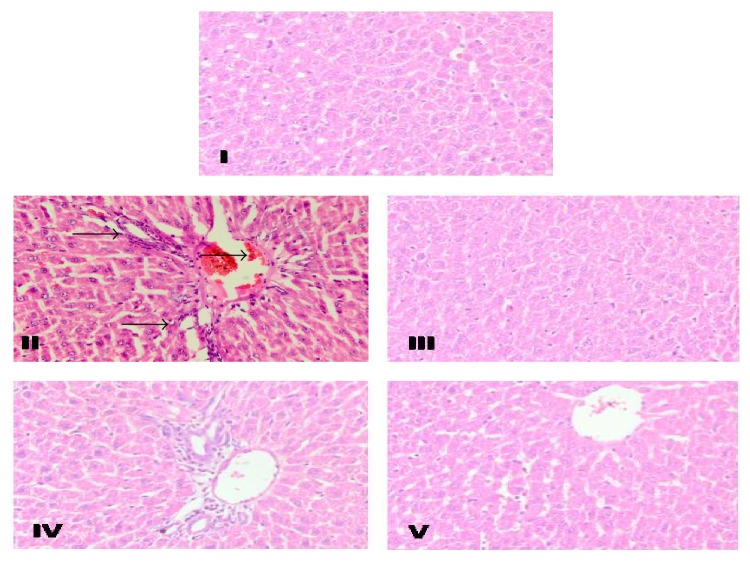
Representative photomicrographs showed effects of* KPEt* on INH-RIF induced histological changes in the rat livers. Representative photomicrographs (magnification ×40). Group I: liver sections treated with 0.9% normal saline showing normal liver architecture. Group II: only INH-RIF induced liver showing portal inflammation, vacuolation, and fatty changes. Group III: liver sections of INH-RIF and Silymarin treated liver showing normal architecture. Group IV: liver sections of INH-RIF and* KPEt* 200 mg/kg b.wt treated group showing mild inflammation and steatosis. Group V: liver sections of INH-RIF and* KPEt* 400 mg/kg b.wt treated showing almost normal architecture.

The histopathological studies revealed that there is change in the normal liver architecture and evident hepatocellular necrosis, congestions of sinusoidal spaces in the centrilobular area, steatosis, and inflammation in group II in comparison to group I. Treatment with Silymarin in group III prevented all the histopathological abnormalities induced by INH-RIF which is in agreement with previous findings of Asha et al. [47]. Group III showed maximum recovery of hepatocytes specifying its significant hepatoprotective activity. Similarly group V also showed marked recovery but recovery was lesser compared to group III and more significant compared to group IV as shown in Figure 2. The hepatic histological changes revealed that the reactive oxygen metabolites and lipid peroxidation could be the reason for different hepatic cellular injuries, that is, centrilobular necrosis, hepatic cell augmentation, steatosis, ballooning degeneration, and periportal fibrosis with impairment of normal liver engineering. The acetylated product of INH, acetylhydrazine, covalently binds to lipid membranes of liver and causes oxidative deterioration of lipids resulting in adipose tissue displacement in the hepatic cells [48]. The photomicrographical investigation of groups III and V shows the recovery of hepatocytes from steatosis, necrosis, and inflammation in comparison to group II.

**Figure 3:**
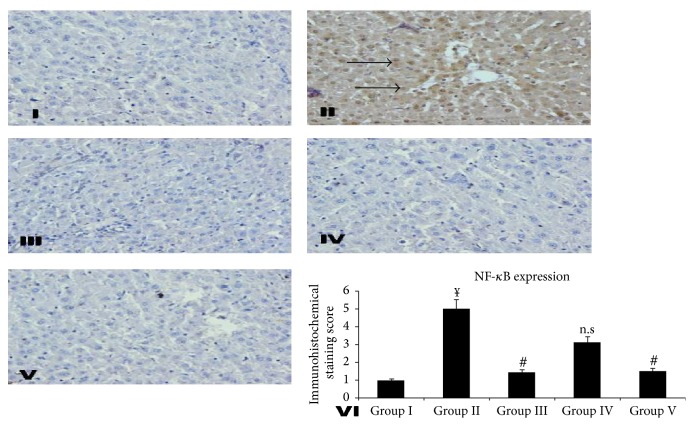
Effect of different doses of* KPEt* on RIF-INH induced NF-*κ*B expression in rat liver. Representative photomicrographs (magnification ×40). Group I: liver sections of 0.9% normal saline treated rats. Group II: hepatic sections of only RIF-INH fed rats showing higher nuclear translocation of NF-*κ*B. Group III: liver sections of RIF-INH and Silymarin treated group showing almost no expressions of nuclear translocation of NF-*κ*B. Group IV: liver sections of RIF-INH and* KPEt *200 mg/kg b.wt treated group showing mild expressions of nuclear translocation of NF-*κ*B. Group V: liver sections of RIF-INH and* KPEt *400 mg/kg b.wt treated group showing less expressions of nuclear translocation of NF-*κ*B. Group VI: scoring data for NF-*κ*B positive cells counted on ten different loci randomly selected on the slide. Values are expressed as ^*¥*^ *P* < 0.001, toxic versus normal, ^*∗*^ *P* < 0.05, extract treated groups versus toxic, ^#^ *P* < 0.01, extract treated groups versus toxic, and ^*¥*^ *P* < 0.001, extract treated groups versus toxic; n.s, not significant.

Conventional immunohistochemistry evaluation of the liver was performed to supplementarily support the biochemical and histopathological examination evidence. The effect of INH-RIF induced NF-*κ*B activation led to maximum nuclear translocation signifying INH-RIF to cause activation of NF-*κ*B. However,* KPEt* treated groups caused a marked attenuation in nuclear translocation as shown in Figure 3. The activation of NF-*κ*B linked regulatory pathways generally underlies inflammatory processes, and an increase in the nuclear translocation of NF-*κ*B has been demonstrated [49]. The transcription factor NF-*κ*B helps to regulate the expression of several genes activated during inflammation and is implicated in several phenomenon such as cellular proliferation and preclusion of apoptosis [50].

**Figure 4:**
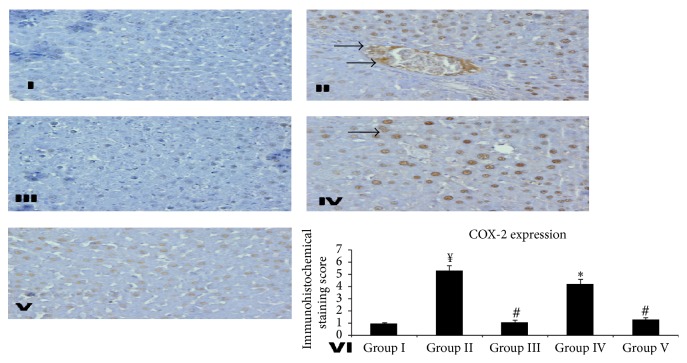
Effect of different doses of* KPEt* on RIF-INH induced COX-2 expression in rat liver. Representative photomicrographs (magnification ×40). Group I: liver sections of 0.9% normal saline treated rats. Group II: hepatic sections of only RIF-INH fed rats showing higher expression of COX-2 as brown patches. Group III: liver sections of RIF-INH and Silymarin treated group showing almost no expressions. Group IV: liver sections of RIF-INH and* KPEt *200 mg/kg b.wt treated group showing moderate expressions. Group V: liver sections of RIF-INH and* KPEt *400 mg/kg b.wt treated group showing mild expressions. Group VI: scoring data for COX-2 positive cells counted on ten different loci randomly selected on the slide. Values are expressed as ^*¥*^ *P* < 0.001, toxic versus normal, ^*∗*^ *P* < 0.05, extract treated groups versus toxic, ^#^ *P* < 0.01, extract treated groups versus toxic, and ^*¥*^ *P* < 0.001, extract treated groups versus toxic; n.s, not significant.

Substantial evidence reveals that the activation of NF-*κ*B upregulates the transcription of COX-2 gene which is responsible for the development of inflammatory response. COX-2 is capable of forming the prostaglandin synthase enzymes, through stimulation of the prostaglandin production pathway [51]. The inhibition of COX-2 has been revealed to exert the hepatoprotective effect in liver damage [52]. Brown color clearly indicates the more number of cells having COX-2 expression (hence more damage) in group II when compared with that of group I. Treatment with Silymarin in group III results in markedly reducing the number of cells showing expression of COX-2. However, there was no marked difference observed in the expression of COX-2 in group IV as compared with group II. Group V showed lesser expression of COX-2 compared to group II. We observed that there is decrease in COX-2 expression by Silymarin and* KPEt* (group V only) which indicates inhibition of prostaglandin synthesis and amelioration of the inflammatory reaction Figure 4.

## 4. Conclusions

In the present study,* KPEt* showed protective effect against INH-RIF induced hepatocellular damage by inhibiting oxidative stress, maintaining balance of antioxidant (enzymatic and nonenzymatic) and distinct decline in COX-2 and NF-*κ*B expressions in rodents. The hepatoprotective capacity of* KPEt* is possibly because of free radical scavenging and antioxidant property resulting from the presence of flavonoids and phenolic acids in the extract as analyzed by using UHPLC-DAD-QToF-MS.

## Figures and Tables

**Table 1:** Characterization of chemical constituents of *KPEt* using UHPLC-DAD-QToF-MS.

Peak number	*R*_*t*_ (min)	Exact mass	*λ*_max⁡_ observed (nm)	Mass observed [M + H]^+^ (*m*/*z*)	Mass observed [M − H]^−^ (*m*/*z*)	Molecular formula	Compound name
1	3.14	354.0945	295 sh, 325	355.1021 (355.1024)	353.0874 (353.0878)	C_16_H_18_O_9_	Chlorogenic acid^1,3^
2	4.18	180.0417	324, 298	181.0495 (181.0495)	179.0351 (179.0350)	C_9_H_8_O_4_	Caffeic acid^ 1,2,3^
3	6.55	194.0574	324, 298	195.0654 (195.0652)	193.0506 (193.0506)	C_10_H_10_O_4_	Ferulic acid^1,2,3^
4	10.85	286.0472	277	287.0552 (287.0550)	285.0403 (285.0405)	C_15_H_10_O_6_	Luteolin^1,2,3^
5	10.99	302.0421	256, 372	303.0496 (303.0499)	301.0354 (301.0354)	C_15_H_10_O_7_	Quercetin^2,3^
6	12.93	272.0679	289, 252	273.0759 (273.0757)	271.0615 (271.0612)	C_15_H_12_O_5_	Naringenin^3^
7	13.06	270.0523	267, 338	271.0606 (271.0601)	269.0454 (269.0455)	C_15_H_10_O_5_	Apigenin^2,3^
8	13.40	286.0472	266, 366	287.0554 (287.0550)	285.0404 (285.0405)	C_15_H_10_O_6_	Kaempferol^2,3^
9	18.41	254.0574	268, 314 sh	255.0658 (255.0652)	253.0507 (253.0506)	C_15_H_10_O_4_	Chrysin^2,3^
10	18.83	256.073	290, 330 sh	257.0811 (257.0808)	255.0662 (255.0663)	C_15_H_12_O_4_	Pinocembrin^2,3^
11	18.99	270.0523	265, 300 sh, 358	271.0605 (271.0601)	269.0461 (269.0455)	C_15_H_10_O_5_	Galangin^2,3^
12	19.51	314.0785	294, 332 sh	315.0864 (315.0863)	313.0719 (313.0718)	C_17_H_14_O_6_	Pinobanksin acetate^2,3^

*R*_*t*_: retention time.

^1^Confirmed with reference; ^2^confirmed with fragmentation pattern; ^3^confirmed with [17–19].

**Table 2:** IC_50_ (half maximal inhibitory concentration)  *in vitro* antioxidant values of propolis from Kashmir Himalaya.

	IC_50_ (*µ*g/mL)	*R* ^2^	Reference	IC_50_ (*µ*g/mL)	*R* ^2^
DPPH scavenging	52.16 ± 6.39	0.985	Vitamin C	11.28 ± 5.23	0.998
NO^•^ scavenging	74.62 ± 5.23	0.973	Chrysin	51.02 ± 3.63	0.989
O_2_^•−^ scavenging	34.77 ± 4.23	0.991	Quercetin	42.54 ± 5.10	0.997

Each value was presented as the mean ± S.E.M; *n* = 3.

**Table 3:** Effect of different doses of propolis on serum parameters in INH-RIF induced hepatotoxicity.

	AST (IU/L)	ALT (IU/L)	ALP (IU/L)	T.bil (mg/dL)	TP (gm/dL)	CHL (gm/dL)	TG (gm/dL)
Group I	145.9 ± 3.391	166.5 ± 2.939	339.9 ± 3.304	0.845 ± 0.385	8.260 ± 0.449	70.75 ± 3.154	57.98 ± 2.297
Group II	303.4 ± 3.654^*¥*^	359.7 ± 3.269^*¥*^	515.3 ± 2.191^*¥*^	2.017 ± 0.497^*¥*^	4.713 ± 0.169^*¥*^	271.4 ± 4.335^*¥*^	165.3 ± 3.036^*¥*^
Group III	176.9 ± 3.588^#^	205.6 ± 2.802^#^	379.6 ± 3.747^#^	1.103 ± 0.049^#^	6.468 ± 0.177^#^	107.1 ± 3.037^#^	93.31 ± 2.400^#^
Group IV	234.2 ± 3.346^#^	271.7 ± 3.036^#^	445.4 ± 3.739^#^	1.690 ± 0.025^#^	6.125 ± 0.212^$^	195.0 ± 3.519^#^	151.8 ± 3.434^*∗*^
Group V	290.1 ± 2.959^*∗*^	341.2 ± 2.737^$^	498.0 ± 3.523^$^	1.830 ± 0.043^*∗*^	4.263 ± 0.870^ns^	256.0 ± 3.587^*∗*^	158.4 ± 2.860^ns^

Values are mean ± S.E.M; *n* = 6; ^*¥*^ *P* < 0.001, toxic versus normal, ^*∗*^ *P* < 0.05, extract treated groups versus toxic, ^$^ *P* < 0.01, extract treated groups versus toxic, and ^#^ *P* < 0.001, extract treated groups versus toxic; ns, not significant.

**Table 4:** Effect of different doses of propolis on *in vivo* antioxidant enzymes in INH-RIF induced hepatotoxicity.

	GPx (nmol NADPH oxidized/min/mg protein)	XO (mg of uric acid formed/min/mg protein)	SOD (units/mg protein)	CAT (nmol H_2_O_2_ consumed/min/mg protein)	MDA (nmol of MDA formed/g tissue)
Group I	189.2 ± 3.877	2.072 ± 0.642	11.56 ± 0.422	4.707 ± 0.345	9.730 ± 0.567
Group II	90.04 ± 2.742^*¥*^	0.978 ± 0.586^*¥*^	7.237 ± 0.323^*¥*^	1.390 ± 0.338^*¥*^	21.31 ± 0.931^*¥*^
Group III	128.2 ± 2.710^*¥*^	1.743 ± 0.780^*¥*^	9.012 ± 0.323^$^	2.718 ± 0.333^#^	15.62 ± 0.512^*¥*^
Group IV	109.1 ± 2.247^#^	1.892 ± 0.894^$^	8.098 ± 0.291^ns^	2.663 ± 0.109^#^	17.95 ± 0.484^*∗*^
Group V	102.6 ± 1.562^*∗*^	1.842 ± 0.507^#^	8.833 ± 0.211^*∗*^	2.260 ± 0.120^*∗*^	16.80 ± 0.948^$^

Values are mean ± S.E.M; *n* = 6; ^*¥*^ *P* < 0.001, toxic versus normal, ^*∗*^ *P* < 0.05, extract treated groups versus toxic, ^$^ *P* < 0.01, extract treated groups versus toxic, and ^#^ *P* < 0.001, extract treated groups versus toxic; ns, not significant.
